# Linking LEDGF/p75 Overexpression With Microsatellite Instability and KRAS Mutations: A Small-Scale Study in Colorectal Cancer

**DOI:** 10.1177/10732748251313499

**Published:** 2025-02-18

**Authors:** Victoria Liedtke, Thomas Wartmann, Wenjie Shi, Ulf D. Kahlert

**Affiliations:** 1 Faculty Environment and Natural Sciences, 38938 Brandenburg University of Technology Cottbus-Senftenberg, Senftenberg, Germany; 2 Molecular and Experimental Surgery, University Clinic for General-, Visceral-, Vascular- and Trans-Plantation Surgery, 61074 Medical Faculty University Hospital Magdeburg, Otto-von Guericke University, Magdeburg, Germany

**Keywords:** LEDGF/p75, colorectal cancer, biomarker, personalized therapy, DNA repair

## Abstract

**Introduction:**

Colorectal cancer (CRC) ranks third in men and second in women, with 153,020 new cases and 52,550 deaths in 2023, and with a projected incidence of 2.2 million new cases by 2030 due to lifestyle changes and enhanced diagnostic capabilities. Identification and analysis of new biomarkers, like lens epithelium-derived growth factor splice variant of 75 kDa (LEDGF/p75), which is known to play a crucial role as stress-related oncogene, can make a significant contribution in facilitating early CRC detection.

**Methods:**

This study analyzed the expression of LEDGF/p75 and the ubiquitin E2 conjugating enzyme UBC13 in 15 CRC tissue samples and adjacent non-tumor tissues. All patient samples underwent NGS-based mutation analysis beforehand. The western blot technique was used for protein analysis, and the results were further validated using mRNA expression data from 521 patient samples from the TCGA database.

**Results:**

LEDGF/p75 expression was significantly elevated in nearly all tumor tissue samples compared to adjacent tissue (11/15, 73.3%). Additionally, the UBC13 enzyme, a key regulator in the degradation of signaling molecules, was also increased in most tumor tissue samples (9/15, 60.0%). Co-overexpression of LEDGF/p75 and UBC13 was evident in 6/6 patients. Patients with KRAS and MSH2 mutations showed a 75% and 100% correlation with LEDGF/p75 overexpression, respectively.

**Conclusion:**

This study confirms the upregulation of LEDGF/p75 in CRC and shows its correlation with KRAS and MSH2 mutations. The interaction of LEDGF/p75 with DNA damage response proteins may contribute to drug resistance and increased tumor aggressiveness. LEDGF/p75’s potential as a prognostic biomarker independent of lymph node involvement or CEA levels highlights its potential in personalized therapy, and warrants further research into its therapeutic targeting.

## Plain language summary

**Introduction:**

Colorectal cancer is a big problem, especially for older adults. It’s the third most common cancer in men and the second most common in women. In 2023, there were over 153,000 new cases and more than 52,000 deaths. In a human body, specific proteins act as biomarkers and can be used to detect the presence of cancer cells (diagnostic biomarker) or cancer progression (prognostic biomarker). Analyzing a new possible biomarker like LEDGF/p75 could help to treat colorectal cancer better.

**Methods:**

In our study, we determined the expression level of the protein LEDGF/p75, a protein that protects cells from stress and damage, and UBC13, which contributes to uncontrolled cell growth, in 15 samples of colorectal cancer tissue and compared it to the level in normal tissue from the same patients. The patient tissue was beforehand checked for common mutations by sequencing the DNA. We also analyzed the production of LEDGF/p75 mRNA of 521 patient samples from a database.

**Results:**

We found that LEDGF/p75 was much higher in the cancer tissue (11 out of 15, or 73.3%). Additionally, expression of UBC13 was also higher in most cancer tissues (9 out of 15, or 60.0%). And in every patient with high LEDGF/p75 levels, UBC13 levels were also high.

**Conclusion:**

This study shows that LEDGF/p75 is more active in colorectal cancer and is linked to certain gene mutations (KRAS and MSH2). LEDGF/p75 helps cancer cells survive damage from treatments like chemotherapy by boosting DNA repair. This can backfire by making them more resistant to these treatments, allowing them to keep growing. So, if we find a lot of LEDGF/p75, it could mean the cancer is more aggressive, and doctors can plan better treatments.

## Introduction

Colorectal cancer (CRC), ranking the third most common cancer in men and the second in women, recorded 153,020 new cases and 52,550 deaths in 2023.^1^ While predominantly affecting people >65 years, 13% of cases occur in individuals under 50 years.^1^

Since 1991, the incidence of CRC has continued to rise as a consequence of lifestyle changes (obesity, diet, low physical activity), environmental changes and demographic factors but also based on enhanced diagnostic capabilities. The efficacy of screening programs, which are primarily based on age and specific biomarkers for CRC, has led to an increase in the reported incidence of CRC due to earlier detection, rather than a true reduction in its occurrence.^2^ The projected burden by 2030 is 2.2 million new cases and 1.1 million deaths, with patients >75 years constituting 44% of the new cases.^3^ Colorectal cancer represents 8.5% of global cancer deaths, necessitating early diagnosis. Screening programs like FOBT (fecal occult blood test) and colonoscopy reduced mortality by 16%,^4^ yet approximately 70% of cases are diagnosed at advanced stages, highlighting the need for enhance early screening to improve survival and life quality.^5^ Unfortunately, current CRC screening programs use a person’s age as a major criterion to initiate screening.^6,7^ The implementation of a comprehensive risk stratification system that considers factors beyond age, coupled with the incorporation of additional demographic, lifestyle, and genetic risk factors, has the potential to enhance the efficacy of current strategies and optimize the utilization of available resources, thereby benefiting patients, health care professionals, and health care systems.^8^

To address this, our study focused on lens epithelium-derived growth factor splice variant of 75 kDa protein (LEDGF/p75) expression in CRC. LEDGF/p75, also known as DFS70 (Dense fine speckled autoantigen of 70 kDa) or PSIP1 (PC4 and SFRS1 interacting protein 1) is considered a ubiquitous nuclear transcription co-activator and is overexpressed in different cancers and cancer cells lines. SUMOylation^9^ of LEDGF/p75 regulates its binding of promotor regions of stress-related proteins, enhancing the activation of AKT-signaling and resulting in increased tumor aggressiveness.^9,10^ Although it is known that LEDGF/p75 is involved in the DNA damage response and promotes signaling pathways of proliferation, chemoresistance and migration,^11^ the mechanistic background has not yet been conclusively clarified.

More than 10 years ago Basu and co-workers analyzed the expression of LEDGF/75 in 21 different tumor types and demonstrated elevated LEDGF/p75 mRNA levels in solid colorectal cancer tissues obtained from random samples.^12^ Briefly, Basu and co-workers used the human ‘TissueScan Cancer Survey Panel 96–I’ QPCR array (CSRT-101, OriGene Technologies Inc., Rockville, MD, USA) for in-house analysis of LEDGF/p75 mRNA expression in 96 tissues covering 8 major human cancer with datasets obtained from the Oncomine database (Compendia Biosciences; Ann Arbor, MI, USA; https://www.oncomine.org). The bioinformatic screening of the microarray data banks performed by Basu and co-workers build up the basis for LEDGF/p75 research. The limitations of such a retrospective study are obvious and include the fact that the results may be subject to temporal or spatial variation. In addition, tumor samples taken at different times may have different molecular profiles, as tumors evolve over time through mutations and other genetic changes.^13^ In addition, previous analyses have lacked consistent sampling methods, leading to conflicting or difficult-to-interpret results. Our study aims to compare the expression of LEDGF/p75 in tumor samples and adjacent non-tumor tissue samples taken at the same time and from the same region of the tumor to minimize the variability and ensures that the observed differences or similarities are due to actual biological properties of the tumor and not due to different sampling conditions. By standardizing the collection and comparison of tissue samples at the same time and from the same region of the tumor, our studies can fill gaps in the existing literature and at the same time provide a more accurate basis for assessing tumor heterogeneity, the mechanisms of action of therapies and the development of personalized medicine approaches.

Consequently, the present study analyzed the individual expression patterns of 15 individuals who donated both tumor and adjacent non-tumor tissue. In combination with database-based mRNA analysis, a significant increase in LEDGF/p75 expression in cancer tissue lysates was confirmed, thereby highlighting the potential of LEDGF/p75 as a biomarker for assessing the likelihood of cancer progression.

## Materials and Methods

### Cell Culture

Human epithelial type 2 (HEp-2, (ATCC Cat# CCL-23, RRID:CVCL_1906), passage number 9 - 18) cells were grown up to 80% confluence in DMEM/Ham’s F12 supplemented with 10% FBS (Biowest, Nuaillé, France), 2 mM L-glutamine (Merck Millipore, Massachusetts, USA) and penicillin/streptomycin (Merck Millipore, Massachusetts, USA) in a humidified incubator at 37°C and 5% CO_2_.

### Participants Characteristics and Tissue Samples

All tissue samples from colorectal cancer patients were taken at Magdeburg University Hospital and are listed in Table 1. A total of 15 patients were included in the study, with randomization designed to ensure representation across a broad spectrum of colorectal cancer classifications.

**Table 1.** Patient Characteristics Including Sample Number, Age, CACI (Charlson-Age Comorbidity Index), Diagnosis, UICC (Union for International Cancer Control), Positive for Metastases (pM), MSI (Microsatellite Instability), Mutation Analysis and LEDGF/p75 Expression in Tumor Tissue.

Sample number	Gender	Age (OP day)	CACI	Diagnosis	UICC	pM	MSI	Mutation analysis	LEDGF/p75 expression in tumor
5669/18	M	87	14	Rectal carcinoma	4C	1	1	KRAS-mut	Increased
2388/18	F	63	6	Rectal adenoma with high grade intraepithelial neoplasia, lynch syndrom	2	0	1	MSH2-germline mutation: c.1566C>G, p.Tyr522Ter	Significant, ***
12950/16	M	70	6	Rectal carcinoma	2A	0	0	0	Significant, **
2765/18	M	64	9	Colorectal carcinoma (sigmoidal colon)	3B	1	0	0	Significant, ***
2227/18	M	67	8	Colorectal carcinoma (right flexure)	4A	1	0	KRAS mut	Significant, *
2714/18	M	65	4	Colorectal carcinoma (ascending colon)	2B	0	0	0	Not increased
7907/17	M	78	6	Colorectal carcinoma (sigmoidal colon)	2A	0	0	0	Not increased
2669/18	M	51	4	Cecal carcinoma	2C	0	0	KRAS mut	Significant, **
13380/16	F	86	8	Cecal carcinoma	3C	0	0	N/A	Increased
9309/18	F	85	10	Cecal carcinoma	4C	1	0	0	Significant, *
1913/19	M	59	7	Colorectal carcinoma (right flexure)	2B	0	1	N/A	Significant, **
3356/17	F	25	3	Colorectal carcinoma (colon transversum), hemicolectomy	2A	0	0	KRAS-mut	Significant, *
4535/18	M	71	6	Colorectal carcinoma (descending colon), hemicolectomy	1	0	0	N/A	Increased
7051/17	F	80	6	Colorectal carcinoma (ascending colon), hemicolectomy	1	0	0	N/A	Significant, ***
12384/18	F	89	7	Rectal carcinoma	2A	0	0	N/A	Increased

### Ethics Approval

This study was performed in line with the principles of the Declaration of Helsinki. Approval was granted by the Ethics Committee of the Medical Faculty of University Hospital Magdeburg (33/01, amendment 43/14).

Informed consent was obtained from all individual participants included in the study. Additionally, the reporting of this study conforms to REMARK guidelines.^14^

### Tissue Sample Preparation

Patient samples (∼50 mg of weight) from tumor and adjacent normal tissue were homogenized on ice in lysis buffer containing 25 mM HEPES (pH 7.5), 75 mM NaCl, 0.5% Triton X-100, 0.5% Nonidet P40, 0.5% Na-deoxycholate, 0.1% SDS, 10% Glycerol and 1 mM EDTA in the presence of different protease inhibitors (10 mM NaF, 20 mM Na4P2O7, 1 mM PMFS, and 1 μg/ml aprotinin) using a Fastprep-24 5G homogenizer (matrix E-tubes, 3 intervals a‘ 30 sec, acceleration of 8 m/sec) and cleared from nuclei and tissue debris by centrifugation at 10.000 ×g for 10 min.

### Antibodies

The following antibodies were used in this study: anti-*γ*H2AX (Cell Signaling Technology, Cat# 2577, RRID:AB_2118010, Massachusetts, USA), anti-N-PSIP1 (Santa Cruz Biotechnology, Cat# sc-101087, RRID:AB_2171222, Texas, USA), anti-UBC13 (Santa Cruz Biotechnology, Cat# sc-376470, RRID:AB_11150503, Texas, USA), anti-mouse-IgGκ BP-HRP (Santa Cruz Biotechnology, Cat# sc-516102, RRID:AB_2687626, Texas, USA).

### Protein Determination

Tissue samples from tumor and adjacent non-tumor were homogenized in lysis buffer and protein content was determined by Bradford assay (#ADV01, Cytoskeleton Inc., Denver, USA).

### Sodium Dodecyl Sulfate (SDS) – PolyAcrylamid Gel Electrophoresis

SDS-page was performed using standard protocols.^11^ Briefly, protein lysate, equivalent to 40 μg protein, was loaded onto an SDS-page with 12% separation gel. Electrophoresis was performed in an Owl™ system (Thermo Fisher Scientific, Massachusetts, US) at 45V for 90 min, followed by 120 V for 100 min.

### Immunoblotting

Immunoblotting was performed, as described elsewhere.^11^ Briefly, protein transfer was performed in a Biometra FastblotTM blotting chamber (Analytik Jena GmbH + Co. KG, Jena, Germany) for 2 h at 50 mA/gel. Membranes were afterwards stained with ponceau S-solution (0.5% (w/v)) to verify protein transfer. Membrane was blocked with 2% milk in TBS-T for 1 h at RT, followed by incubation with primary antibodies for 1 h, RT. Primary antibodies were diluted as follows: anti-N-PSIP1 (1:1000), anti-Ubc13 (1:1000), anti-RPA32 (1:1000) and anti-yH2AX (1:1000)-antibodies were diluted in 2% BSA/TBS/0.1% Tween-20 (Carl Roth, Karlsruhe, Germany), anti-GAPDH (1:10000) antibody was diluted in 2% milk powder/TBS/0.1% Tween-20 (Carl Roth, Karlsruhe, Germany). Secondary antibody (anti-mouse-IgGκ BP-HRP) were diluted 1:5000 in 5% milk powder/TBS/0.1% Tween-20) and incubated for 1 h at RT. The protein bands were visualized using ECL-solution (100 μl of 250 mM Luminol, 100 μl of 90 mM p-coumaric acid, 10 μl H_2_O_2_ (30%) in 20 mL 1 M Tris/HCl, pH 8.5) and intensity was quantified by ImageJ software (1.53c 26). Original images can be found in Supplement 1.

### RNA Sequencing Data

MRNA Sequencing data and related clinical information on colon cancer like CEA-level was obtained from GDC cohort from “The Cancer Genome Atlas Program” (TCGA)^15^ research network and can be downloaded directly from xenabrowser (TCGACOAD.htseq_fpkm.tsv). Datasets are also available on request via Microsoft OneDrive. The patients with a lack of follow-up records were excluded. Finally, a total of 521 mRNA samples were enrolled in this study, including 41 adjacent samples and 480 cancer samples.

### Next-Generation Sequencing

Patient tumor tissue was analyzed towards driver mutations like KRAS, NRAS, PIK3CA, BRAF by using the “AmoyDx® HANDLE Classic NGS Panel” (AmoyDx, Singapore, Asia) according to the manufacturer’s instructions. MSH2 mutation status was analyzed using “Nextera Rapid Capture Custom Enrichment Panel” (Illumina, California, USA).

### Statistical Analysis

All data were analyzed with the statistical computing language R ≥4.2.1.^16^ For the analysis of mRNA data the packages DSeq and limma were used and applied according to their default settings. The corresponding code is available in Suppl. 2. Adjusted *P*-values less than 0.05 were considered as significant. Experiments were conducted with at least 3 biological replicates. For the analysis of band intensity, representing the expression of protein of interest (LEDGF/p75, UBC13, *γ*H2AX and GAPDH) compared to whole protein amount) one-way analysis of variance (ANOVA) and comparison between 2 groups (unequal sample size of tumor and non-tumor tissue) was performed by paired student *t* test. *P*-value of lower than 0.05 was considered statistically significant.

## Results

### Overexpression of LEDGF/p75 in Tumor Tissue Results in Enhanced DNA Damage Response

**Figure 1.**
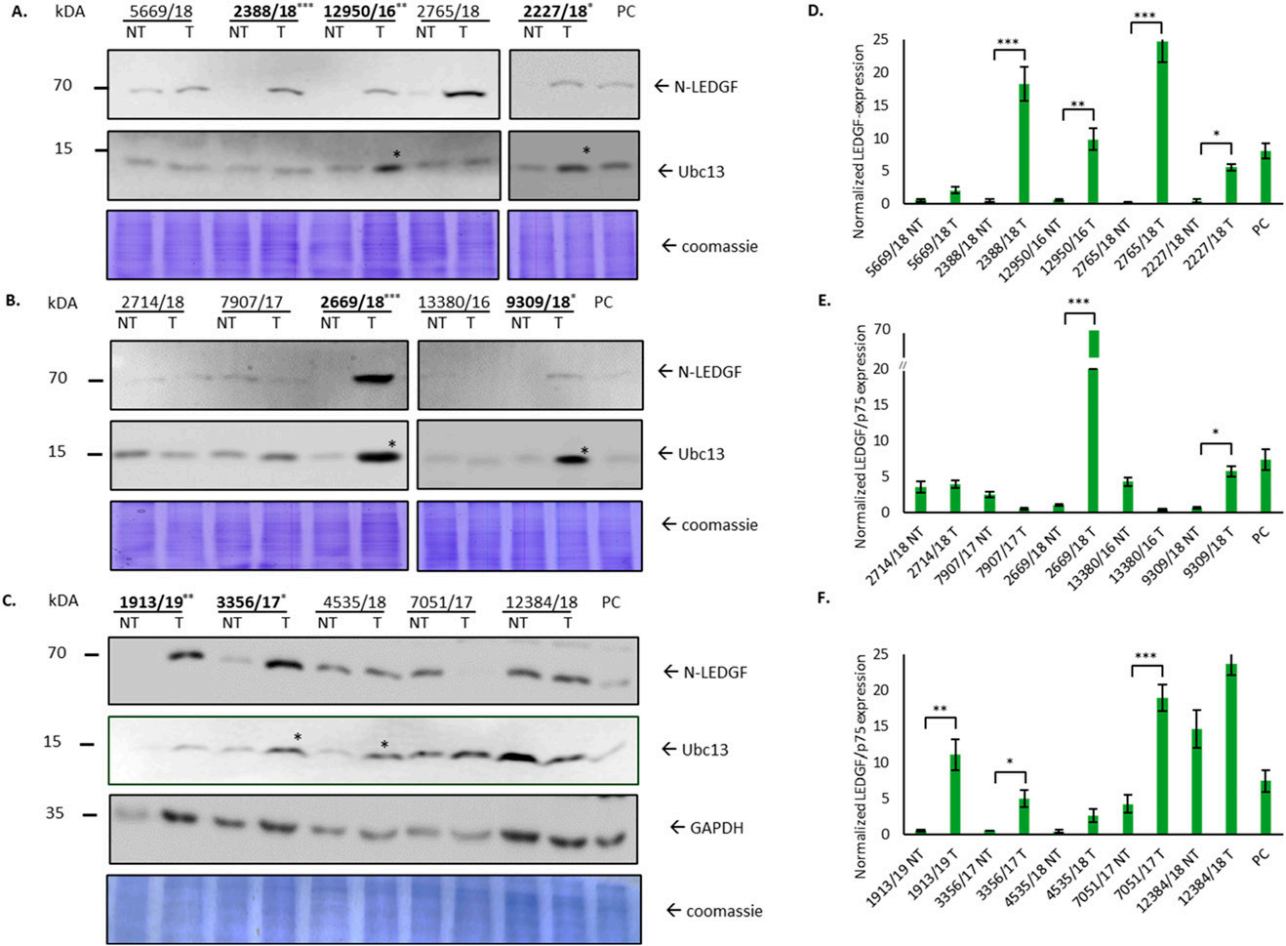
Overexpression of LEDGF/p75 in tumor tissue causes dysregulation in DNA damage response. (A–C) Immunoblot using antibodies against N-terminal LEDGF/p75, Ubc13 and GAPDH on tissue samples from different donors, indicated by numbers and abbreviated with “NT” for non-tumor and “T” for tumor tissue sample. Hep-2 cells were used as positive control (PC). Total protein staining was performed by Coomassie brilliant blue and used as loading control (n = 3). (D–F) Quantitative analysis of LEDGF/p75 expression in different tumor and non-tumor tissue samples, (n = 3), *P < 0.05, **P < 0.01, ***P < 0.001.

LEDGF/p75 and UBC13, identified for their interaction in cell cultures, play crucial roles in DNA repair, transcriptional regulation and cell survival. Investigating their relevance in human colorectal cancer tumor and non-tumor tissues, immunoblotting revealed that increased LEDGF/p75 expression (significant increased expression in 9/15, 60.0%) correlated with elevated UBC13 levels in tumor samples (6/6, 100%) (Figure 1(A)–(C)).

Here, we were able to show that LEDGF/p75 seems to have an indirect or direct effect on regulatory proteins of the DNA damage response pathway such as UBC13, which is known to be involved, eg, in the degradation of the DNA double strand marker γH2AX (phosphorylated histone 2AX).

Notably, GAPDH expression showed donor-dependent variability within the same donor’s samples (Figure 1(C)) and was replaced with whole protein analysis by Coomassie brilliant blue staining. If the total protein content is determined by Coomassie staining and used as the basis for calculating the expression intensity, this problem can be avoided. These findings clearly highlight the potential significance of LEDGF/p75 and UBC13 in cancer contexts.

### Positive Correlation Between LEDGF/p75 Expression and KRAS Mutation in Patient-Derived Tumor Tissue

As genetic alterations may cause tumorigenesis, we analyzed the patient-derived tumor tissues, compared the expression of LEDGF/p75 with tumor mutation analysis of prominent colorectal cancer biomarkers (KRAS, BRAF) and recognized a positive correlation in 75% (3/4) of patients with KRAS mutation and significantly increased LEDGF/p75 expression (Table 1). Furthermore 100% (3/3) of patients with MHS2 germline mutation and microsatellite instability also represent a significantly increased LEDGF/p75 expression in the tumor tissue (Table 1).

### Statistical Analysis Revealed Significantly Increased Expression of LEDGF/p75 mRNA in Colorectal Cancer Tissue

**Figure 2.**
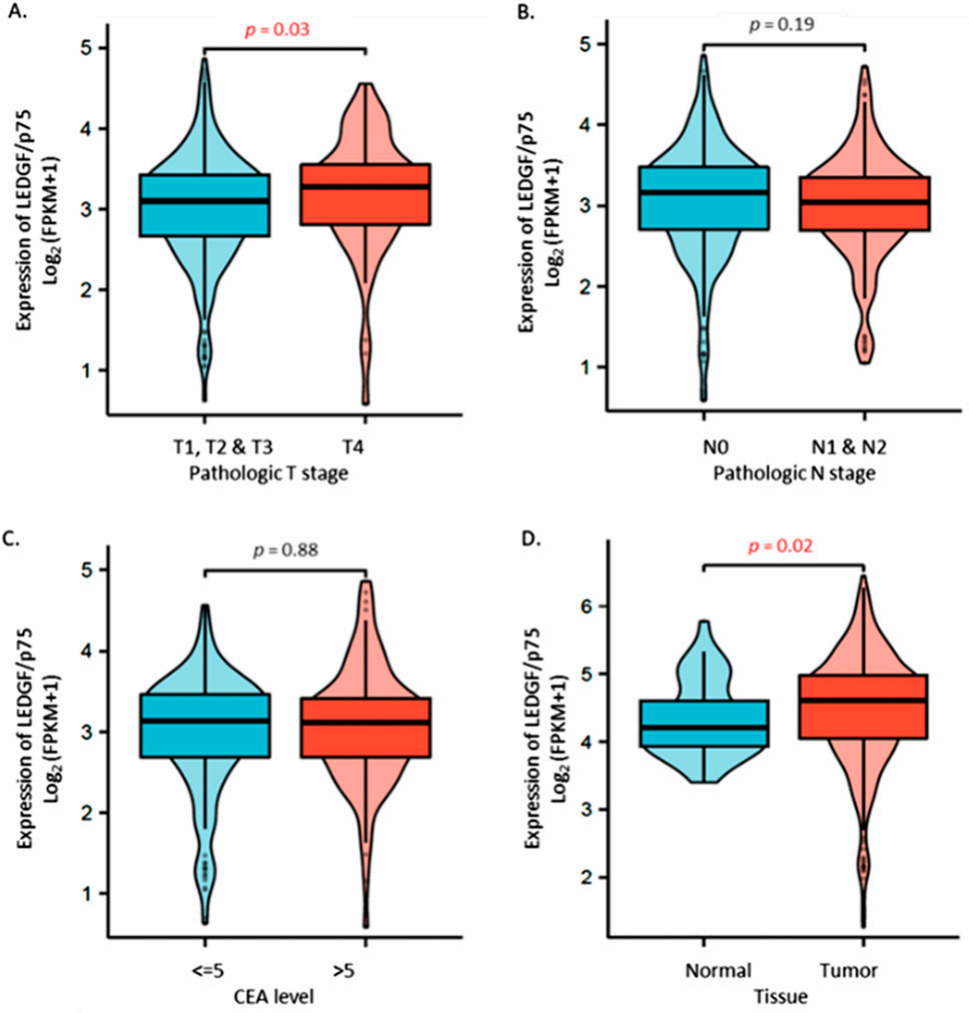
Analysis of LEDGF/p75 mRNA expression towards clinical factors in colorectal cancer tissue. (A) total of 521 patients were enrolled in this study, including 41 adjacent samples and 480 tumor samples. LEDGF/p75 mRNA expression was analyzed towards A. pathologic T stages T1-T3, compared to T4 stage (n_T1_ = 11, n_T2_ = 84, n_T3_ = 327, n_T4_ = 60. (B) pathologic N stages N0, compared to N1 & N2. (C) CEA level and D. between adjacent (normal) and tumor tissue. The equations should be inserted in editable format from the equation editor.

Verification of obtained protein data was performed by bioinformatic-based mRNA analysis of LEDGF/p75 in a cohort of 521 patients including 41 adjacent samples and 480 tumor samples. The subdivision of the sequencing data is based on the TNM Classification of Malignant Tumors with special attention to the T (size or direct extent of the primary tumor) and N (degree of spread to regional lymph nodes) stages. Expectedly, LEDGF/p75 mRNA expression was significantly increased in tumor stage T4 (n = 60), compared to stages T1-T3 (n_T1_ = 11, n_T2_ = 84, n_T3_ = 327; Figure 2(A)). However, there was no difference in LEDGF/p75 mRNA expression in CRC patients with or without pathologically involved lymph nodes, nor did LEDGF/p75 expression show an effect on carcinoembryonic antigen (CEA) - level (Figure 2(B)–(C)). Nevertheless, the significantly increased LEDGF/p75 mRNA expression in tumor tissue compared to non-tumor tissue was clearly demonstrated (Figure 2(D)).

## Discussion

Studies on the expression of LEDGF/p75 in colorectal carcinomas are rare, so our study represents a promising extension of this field. This study confirms the findings of Basu et al. (2012) on the expression of LEDGF/p75 in colorectal cancer tumor and non-tumor tissues. In 15 patients, 60% of tumor samples showed significant upregulation of LEDGF/p75, highlighting individual heterogeneity. The variability of GAPDH expression has been noted in previous cell culture studies where increased GAPDH expression was observed with LEDGF/p75 overexpression.^11^ LEDGF/p75 is known to be critical for genomic integrity, promoting DNA repair and acting as a transcriptional co-activator by expressing oncogenes like Hsp27 under stress conditions.^11,17^ The dual function of LEDGF/p75 as both an oncoprotein and a tumor suppressor through the DNA repair mechanism is a false reciprocity. In reality, the overexpression of LEDGF/p75 leads to the activation of oncogenes, while at the same time the involvement of LEDGF/p75 in DNA repair promotes the growth of these genetically unstable cells. Its interaction with UBC13, previously shown by our group in 2021, may contribute to drug resistance by increasing the dysregulation of DNA damage repair mechanisms.^11^ UBC13 is a ubiquitin-conjugating enzyme that catalyzes the formation of polyubiquitin chains linked through lysine 63 (K63) of ubiquitin. These K63-linked ubiquitin chains differ from the more commonly studied K48-linked chains that target proteins for proteasomal degradation. Instead, K63-linked chains are involved in signaling and recruiting key DNA repair proteins, including RAP80, BRCA1 and 53BP1, to the DNA double-strand break.^18^ Overexpression of LEDGF/p75 may therefore lead to enhanced DNA repair pathways in colorectal cancer.

Interestingly, and despite the small sample size, significantly increased LEDGF/p75 expression correlated in 75% of patients with KRAS mutation and in 100% of patients with MSH2 germline mutation. The correlation of LEDGF/p75 expression with these two proteins is of interest because KRAS mutations are common in early stages of colorectal cancer and are considered to be one of the first genetic mutations in the course of tumor development.^19^ It is therefore essential that future experiments significantly increase the sample size in order to reinforce the validity of our results. In addition to the tumor stage, it is crucial to consider the age of the patients, as the probability of KRAS mutation increases with age.^20^ KRAS mutations lead to the activation of downstream signaling pathways such as the MAPK/ERK and PI3K/AKT pathways, while LEDGF/p75 can enhance the transcriptional activation of genes within these pathways, thereby promoting tumorigenesis and enhancing the growth and survival of KRAS-mutant EGFR inhibitor-resistant cancer cells. Therefore, incorporating LEDGF/p75 detection into prognostic assays, such as blood tests or biopsy analyses, might help identify patients with early-stage colorectal cancer and provide prognostic information by indicating how the tumor is likely to behave and respond to treatment. MSH2 mutations, on the other hand, affect the DNA mismatch repair system, leading to increased microsatellite instability (MSI), an accumulation of DNA errors leading to increased genomic instability and a higher risk of cancer development. While MSH2 mutations and MSI have been demonstrated to result in elevated mutation rates and genomic instability, evidence suggests that LEDGF/p75 overexpression may confer a survival advantage to these genetically unstable cells, thereby facilitating their proliferation and expansion. A deeper comprehension of the interrelationship between MSH2 mutations, MSI and LEDGF/p75 overexpression may offer valuable insights into the development of novel therapeutic strategies. For example, targeting LEDGF/p75 by siRNA treatment or combined immunotherapy in MSI-positive tumors with MSH2 mutations could sensitize these tumors to chemotherapy or increase the efficacy of immunotherapy.

Increased expression of LEDGF/p75 indicates genomic instability, making it a potential prognostic biomarker for colorectal cancer progression. As prognostic biomarkers indicate an increased (or decreased) likelihood of a future clinical event, we therefore hypothesize that an increased expression of LEDGF/p75 correlates with a more aggressive cancer behavior.

In our experiments, significant differences in LEDGF/p75 expression between normal and tumor tissues were validated by western blot and bioinformatic-based mRNA analysis. A comparative analysis of these methodologies reveals notable discrepancies in their relative significance, which will be described in detail in the following section. While a significantly increased expression of LEDGF/p75 in the bioinformatic-based mRNA level analysis could only be detected in stage IV of colorectal cancer, the patient-specific protein analysis showed a significantly increased expression of LEDGF/p75 already detectable in stages I and II of colorectal cancer patients. Higher expression of LEDGF/p75 mRNA was detected regardless of lymph node involvement or expression of carcinoembryonic antigen (CEA) levels. This is not an uncommon occurrence, and both characteristics are suitable for using LEDGF/p75 as prognostic biomarkers. The use of biomarkers that are not dependent on lymph node status could thus provide additional prognostic information and enable the identification of high-risk patients who may require more aggressive treatment. This may assist in the tailoring of treatment plans, the avoidance of over-treatment in patients who may not benefit from certain therapies, and the provision of more aggressive treatment to those who require it. CEA is a glycoprotein involved in cell adhesion and is widely used as a tumor marker in colorectal cancer for prognosis, monitoring treatment response and detection of recurrence. However, CEA levels are known to differ depending on whether the tumor is in the colon or rectum. Studies have shown differences in CEA expression between proximal (right-sided) and distal (left-sided) colorectal tumors. In addition, a patient’s individual genetic background (KRAS mutation, MSI) can also influence CEA expression.^21^ Although elevated CEA levels are generally associated with a poorer prognosis, the variability means that CEA alone cannot be used definitively to predict outcome. It should be interpreted in conjunction with other clinical findings and diagnostic tests. Nevertheless, CEA levels can sometimes influence therapeutic decisions, particularly in the context of metastatic disease.

Combined analysis of LEDGF/p75 expression with CEA levels and KRAS and MSH2 mutation status can significantly improve prognostic accuracy in colorectal cancer patients by providing a more complete understanding of tumor behavior and progression. By integrating these factors, it might be possible to better stratify patients based on their risk of progression, tailor individual treatment plans, and make more informed decisions about therapeutic approaches. This combined and ongoing analysis might also help to monitor disease progression and response to treatment, allowing early detection of relapse and timely intervention.

In further research directions, co-cultivation of genetically modified patient-derived organoids with LEDGF/p75 knock-out and an overexpression of LEDGF/p75, from colorectal cancer patients with autologous immune cells might give insights into LEDGF/p75-dependent immune cell infiltration and therefore tackling the question of immune escape in cancer cells. Additionally, immunocompetent, patient-specific tumor- and non-tumor-microtissues will be generated and treated with allosteric integrase inhibitors (which target LEDGF/p75) as potential therapeutic agents to elucidate the effect of inhibited LEDGF/p75 towards cellular proliferation and migration.

We recognize that this study is not without limitations. For example, the small sample size and the heterogeneity of LEDGF/p75 expression require a larger experimental setting to confirm our results.

## Conclusion

In conclusion, this study contributes to the existing research on LEDGF/p75 expression in colorectal cancer by confirming its upregulation in tumor tissues, thereby corroborating the findings of Basu et al. (2012). This study identifies a correlation between LEDGF/p75 expression, KRAS mutations and MSH2 germline mutations, indicating LEDGF/p75’s involvement in genomic instability. The involvement of LEDGF/p75 in DNA repair mechanisms and its interactions with proteins such as UBC13 may contribute to the development of drug resistance and tumor progression, increasing tumor aggressiveness. In light of the association between LEDGF/p75 and tumor aggressiveness, irrespective of lymph node involvement or CEA levels, the potential of LEDGF/p75 as a prognostic biomarker is emphasized. Further research is required to validate these findings and to investigate the potential of LEDGF/p75 as a therapeutic target.

## Supplemental Material

Supplemental Material - Linking LEDGF/p75 Overexpression With Microsatellite Instability and KRAS Mutations: A Small-Scale Study in Colorectal Cancer Supplemental Material for Linking LEDGF/p75 Overexpression With Microsatellite Instability and KRAS Mutations: A Small-Scale Study in Colorectal Cancer by Victoria Liedtke, Thomas Wartmann, Wenjie Shi, and Ulf D. Kahlert in Cancer Control

Supplemental Material - Linking LEDGF/p75 Overexpression With Microsatellite Instability and KRAS Mutations: A Small-Scale Study in Colorectal Cancer Supplemental Material for Linking LEDGF/p75 Overexpression With Microsatellite Instability and KRAS Mutations: A Small-Scale Study in Colorectal Cancer by Victoria Liedtke, Thomas Wartmann, Wenjie Shi, and Ulf D. Kahlert in Cancer Control

Supplemental Material - Linking LEDGF/p75 Overexpression With Microsatellite Instability and KRAS Mutations: A Small-Scale Study in Colorectal Cancer Supplemental Material for Linking LEDGF/p75 Overexpression With Microsatellite Instability and KRAS Mutations: A Small-Scale Study in Colorectal Cancer by Victoria Liedtke, Thomas Wartmann, Wenjie Shi, and Ulf D. Kahlert in Cancer Control

## Appendix

### Abbreviations

BSA: bovine serum albumin
CACI: Charlson-Age Comorbidity Index
CEA: carcinoembryonic antigen
CRC: colorectal cancer
DFS70: dense fine speckled autoantigen of 70 kDa
FOBT: fecal occult blood test
H2AX: histone 2 AX
LEDGF/p75: lens epithelium-derived growth factor of 75 kDa
MSI: microsatellite instability
NT: non-tumor
pM: positive for metastasis
PSIP1 PC4 and SFRS1: interacting protein 1
T: tumor
UICC: Union for International Cancer Control
γH2AX: phosphorylated histone 2 AX
