# Supplementary material for: Linking LEDGF/p75 Overexpression With Microsatellite Instability and KRAS Mutations: A Small-Scale Study in Colorectal Cancer
Source: Cancer Control. 2025 Feb 18;32:10732748251313499. doi: 10.1177/10732748251313499 (PMC11837075; doi:10.1177/10732748251313499)
Supplement: Supplemental Material - Linking LEDGF/p75 Overexpression With Microsatellite Instability and KRAS Mutations: A Small-Scale Study in Colorectal Cancer [file sj-pdf-1-ccx-10.1177_10732748251313499.pdf]

Linking LEDGF/p75 Overexpression with Microsatellite Instability and KRAS Mutations: A Small-Scale Study in Colorectal Cancer

[illegible] Cancer Control[illegible]

[illegible] Xenabrowser: https://xenabrowser.net/datapages/?dataset=TCGACOAD.htseq_fpkm.tsv&host=https%3A%2F%2Fgdc.xenahubs.net&removeHub=https%3A%2F%2Fxena.treehouse.gi.ucsc.edu%3A443

Kind regards

Supplement 1:
Original westernblot images to figure 1 A-C

Figure 1 A.

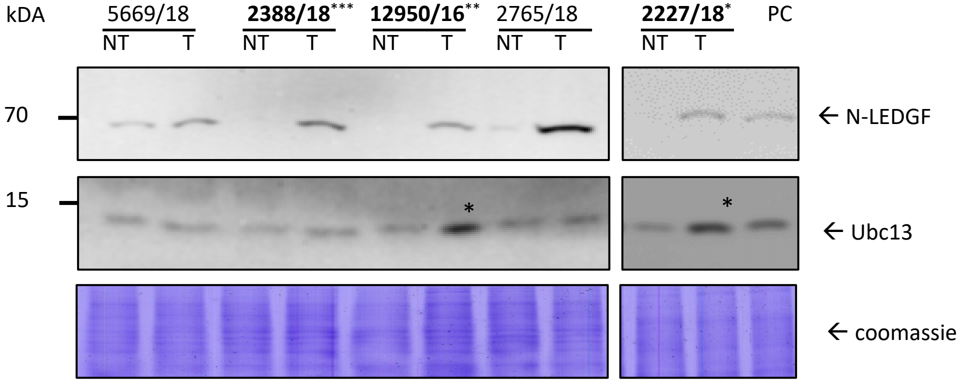

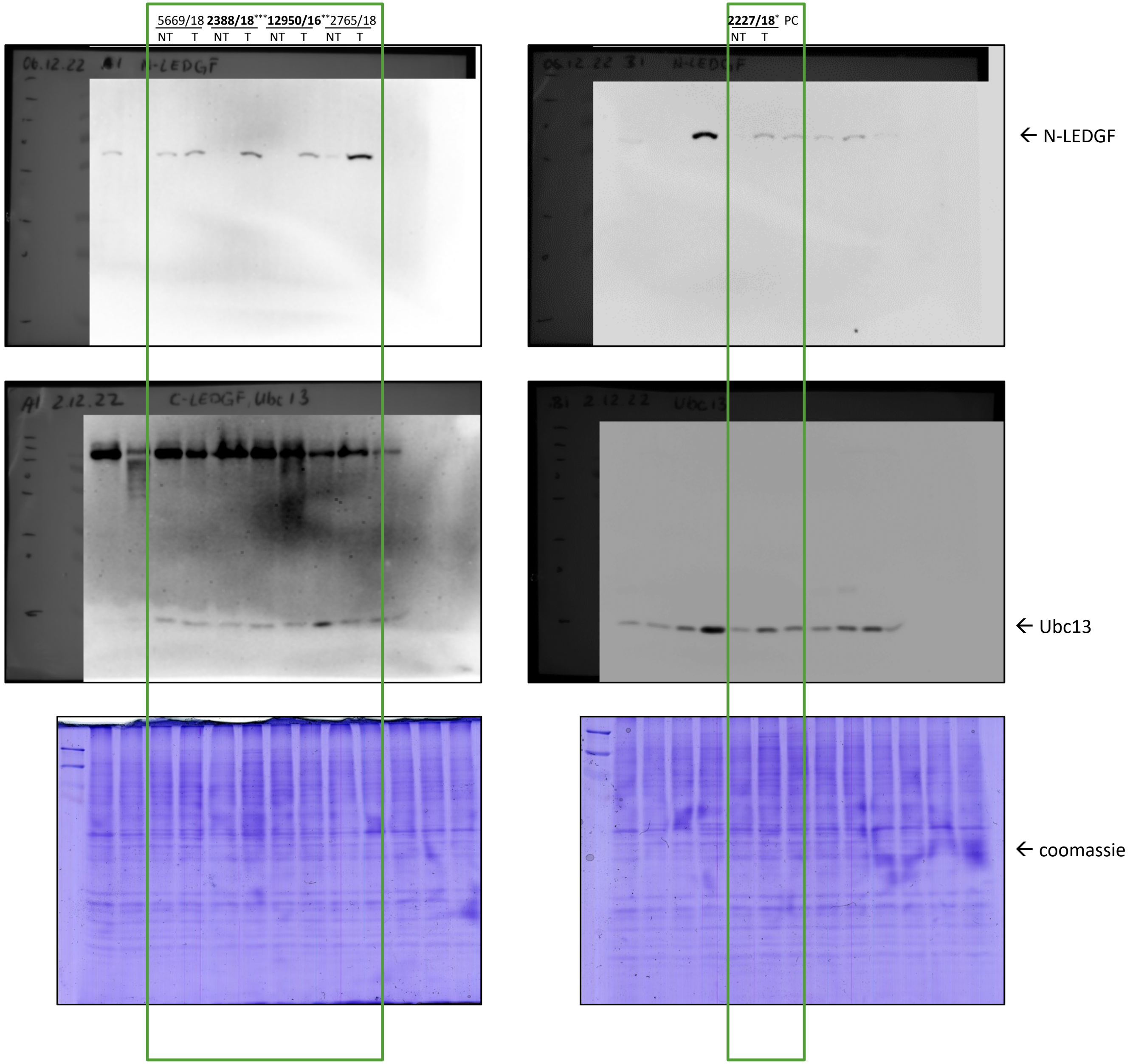

Original westernblot images to figure 1 A-C

Figure 1 B.

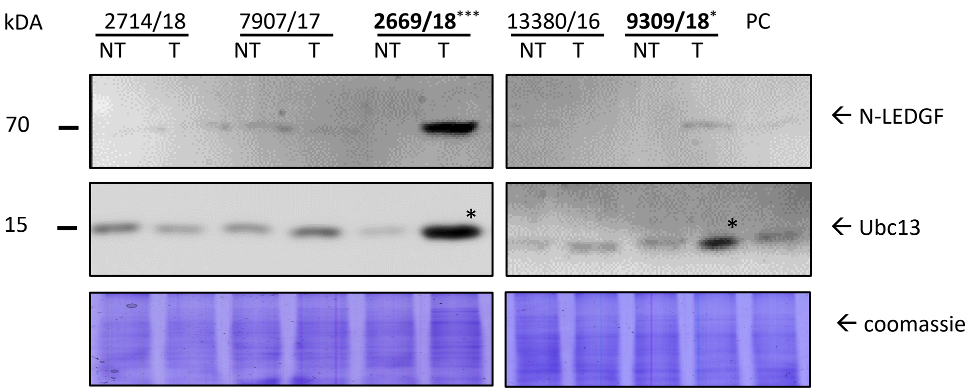

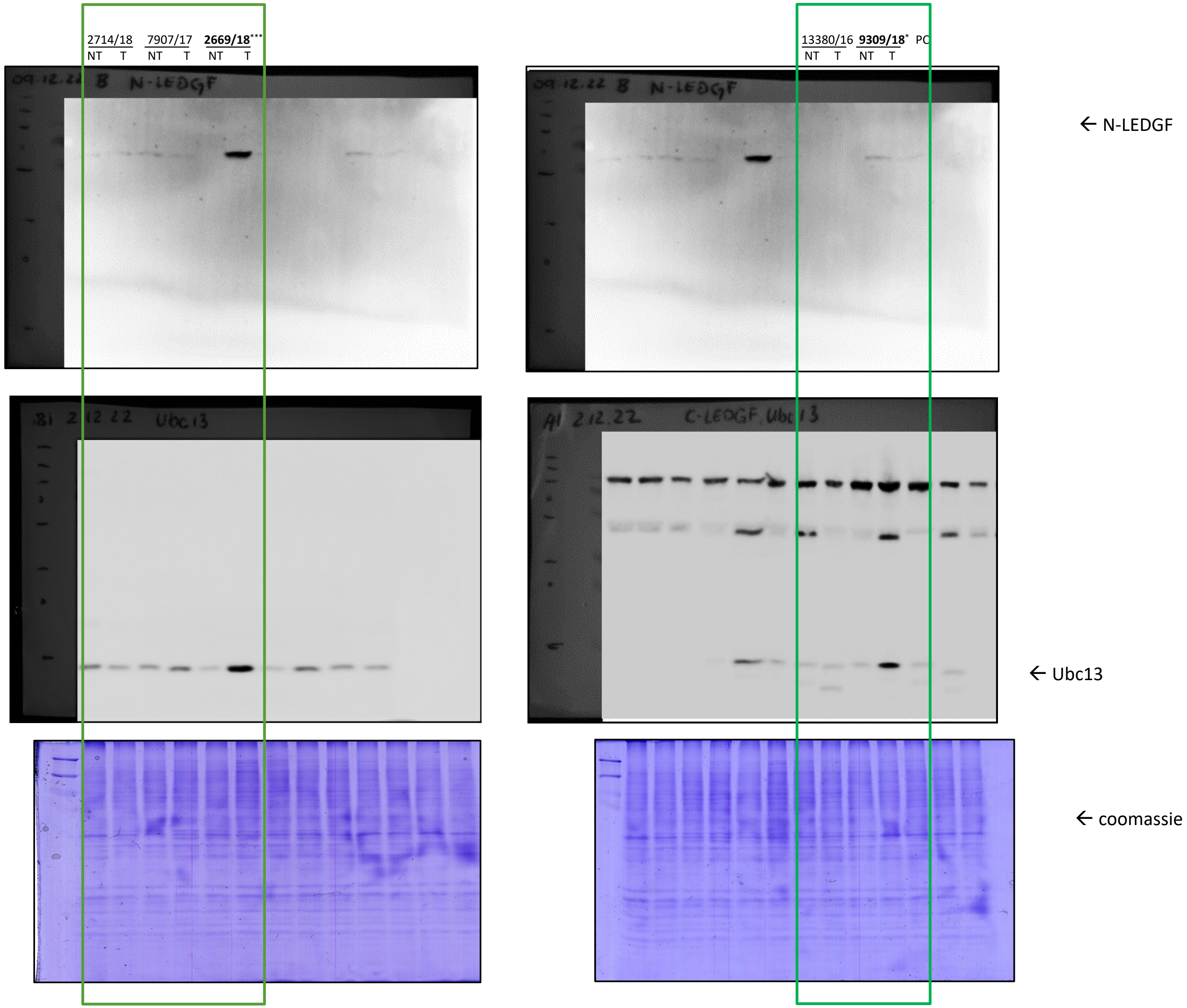

Original westernblot images to figure 1 A-C

Figure 1 C.

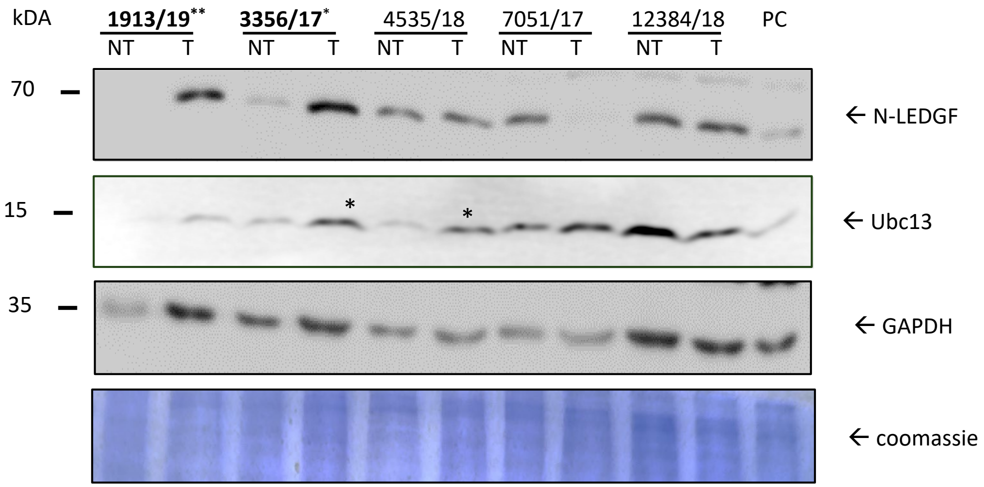

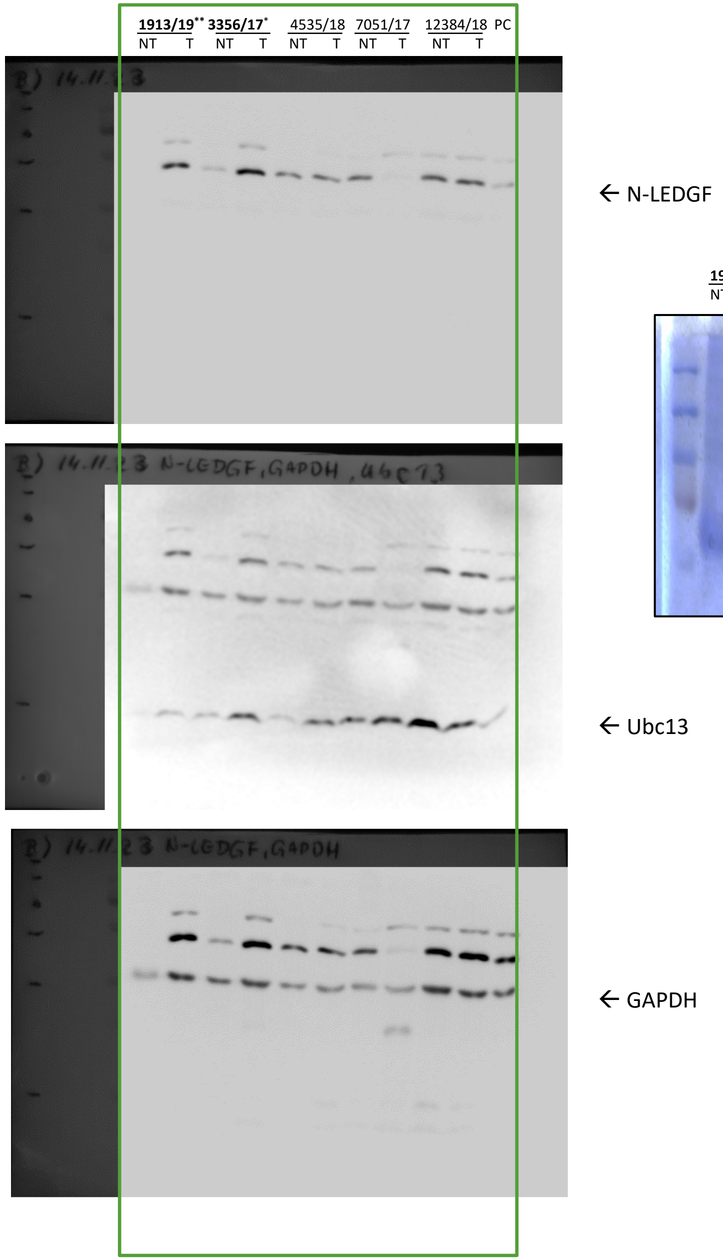

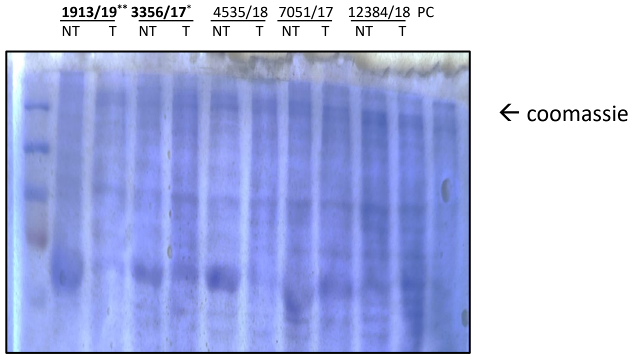

Supplement H:

```
###loading Packages
library(DESeq2)
library(tidyverse)
library(limma)
library(ComplexHeatmap)
library(circlize)
count<-read.table("input.txt",sep="\t",header=T,row.names=1)
count[is.na(count)] <- 0
count<-ceiling(count)
condition <- c(rep("normal",x), rep("tumor",y))
group=as.data.frame(condition)
all(rownames(group) == colnames(count1))
group$condition <- factor(group$condition)
group$condition <- relevel(group$condition, "normal")
DEGs <- DESeqDataSetFromMatrix(count1, group, design = ~ condition)
DEGs <- DESeq(DEGs)
results <- results(DEGs, contrast = c('condition', 'tumor', 'normal'))
results = results[order(results$pvalue),]
results=as.data.frame(results)
results_clean <- na.omit(results)
write.csv(results_clean,file="C_vs_T_DFF_ALL.csv",quote = FALSE)
results_clean[which(results_clean$log2FoldChange >= 1 & results_clean$pvalue <
0.05),'sig'] <- 'up'
results_clean[which(results_clean$log2FoldChange <= -1 & results_clean$pvalue <
0.05),'sig'] <- 'down'
results_clean[which(abs(results_clean$log2FoldChange) <= 1 | results_clean$pvalue >=
0.05),'sig'] <- 'none'

diffgene <- subset(results_clean, sig %in% c('up', 'down'))
write.csv(diffgene,file=" C_vs_T_DIFF.csv")

#Volcano plot
library(ggplot2)
library(ggrepel)
dat<-results_clean
pdf("C_vs_T_volcano_plot.pdf",height=5,width=7)
ggplot(dat,aes(x=log2FoldChange,y=-log10(pvalue),color=sig))+
  geom_point()+
  scale_color_manual(values=c("#2f5688","#BBBBBB","#CC0000"))+   theme_bw()+  theme(
    legend.title = element_blank()   )+
  theme(axis.title.x =element_text(size=14,face = "bold"),
axis.title.y=element_text(size=14,face = "bold"),axis.text = element_text(size =
14,face = "bold")) +   ylab('-log10 (pvalue)')+   xlab('log2 (FoldChange)')+
geom_vline(xintercept=c(-1,1),lty=3,col="black",lwd=0.5) +  geom_hline(yintercept = -
log10(0.05),lty=3,col="black",lwd=0.5)
dev.off()

###heatmap##
rld <- rlog(DEGs)
vsd_df<- as.data.frame(assay(rld))
heatexp=vsd_df[rownames(diffgene),]
filtered_data <- diffgene %>% filter(pvalue < 0.05) %>% arrange(desc(log2FoldChange))
n <- nrow(filtered_data)
top_20_DEG <- rbind(filtered_data[1:10, ], filtered_data[(n-9):n, ])
top_20_heat=heatexp[rownames(top_20_DEG),]
group_data=as.data.frame(group)
colnames(group_data)[1] <- "group"
rownames(group_data)<-colnames(vsd_df)
ann_colors = list(group = c(normal = "#99CC99", tumor = "#993333"))
group_order <- c( "normal", "tumor")
top_20_heat <- top_20_heat[, order(match(group_data$group, group_order))]
pdf("C_vs_T_heat_plot.pdf",height=4,width=6)
pheatmap(top_20_heat,
         annotation_col = group_data,
         annotation_colors = ann_colors,
         show_colnames = T,
         cluster_cols = F,
         fontsize = 7,
         scale="row",
```

```
         gaps_col =x,
         cutree_rows = 2,
         cutree_cols = 2,
         fontsize_row = 6,
         fontsize_col = 6,
         cellwidth = 5,
         cellheight =5)
dev.off()

###boxplot####
mydata<-filtered_data %>%
  gather(key="gene",value="Expression",2:n) %>%
  ##
  dplyr::select(id,gene,Expression,everything())
head(mydata)
mydata$gene <- fct_inorder(mydata$gene)
p1<-ggplot(mybox, aes(x = gene, y = Expression,fill=gene)) +
  geom_boxplot(outlier.colour="black",outlier.size=0.05)
p1
p1+geom_jitter(shape=16,
position=position_jitter(0.2),size=0.1)+theme_light()+theme(axis.text.x =
element_text(angle = 45, hjust = 1))
p1+geom_jitter(shape=16,
position=position_jitter(0.2),size=0.1)+theme_light()+theme(axis.text.x =
element_text(angle = 45, hjust = 1))+theme(legend.position="lef") +
  guides(fill=guide_legend(nrow=2))
```
