# Supplementary material for: Linking LEDGF/p75 Overexpression With Microsatellite Instability and KRAS Mutations: A Small-Scale Study in Colorectal Cancer
Source: Cancer Control. 2025 Feb 18;32:10732748251313499. doi: 10.1177/10732748251313499 (PMC11837075; doi:10.1177/10732748251313499)
Supplement: Supplemental Material - Linking LEDGF/p75 Overexpression With Microsatellite Instability and KRAS Mutations: A Small-Scale Study in Colorectal Cancer [file sj-pdf-3-ccx-10.1177_10732748251313499.pdf]

Data Collection and Presentation Checklist:

**Ethical Statements (if applicable):**		
**Humans**	Complete and upload the Ethics Declaration Statement you received with your decision letter.	X
**Animals**	Provide species, sex, strain, age, source and husbandry conditions.	N/A
	Note if the study was blinded or not.	N/A
	Provide a statement confirming the research was approved by the Institutional Animal Care and Use Committee.	N/A
	Complete and upload the ARRIVE Compliance Questionnaire. Visit ARRIVE for more information.	N/A
**Reagents and Biological Materials:**		
	Include manufacture name, catalog number (and lot number for antibodies) for all reagents used (including fluorochromes and stains).	X
	Cell lines: provide source, derivation and authentication method.	X
**Images**		
**General**	Do not introduce or remove any features in your images. Leave any blemishes.	X
	If any adjustments to contrast, balance or brightness are made, they must be applied uniformly across the entire image. Any nonlinear adjustments must be disclosed in the legend.	X
	Check that images are not pixelated when reasonably magnified. Images must be at 300 dpi. TIFF images are encouraged. Avoid jpegs or using PowerPoint as this will compress your images.	X
	Scale bars must be included.	N/A
	If splicing images, the borders must be marked and noted in the legend.	X
**Microscopy (include the following)**	Camera make and model.	N/A
	Microscope make and model.	N/A
	Objective magnification, type and numerical aperture. Magnification must be mentioned in figure legend.	N/A

	Fluorochromes and stains. They should also be mentioned in the legend.	N/A
	Acquisition software.	N/A
	Show all individual channels in grey scale and merged image in color (all at the same intensity).	N/A
**Western Blots**	Westerns should not be modified for contrast, the entire tonal range should be present.	X
	Include at least two molecular weight markers, one above and one below your band of interest.	X
	If a blot is spliced together, you must mark the border and explain this in the legend. Splicing across different blots is not allowed.	X
	It is best practice to normalize protein levels against total protein, not house-keeping proteins.	X
	Post-translationally modified proteins (PTMs) must use total protein for normalization.	N/A
	Provide raw blots as supplementary data. These may be combined as a single Word doc. Blots must be accurately labeled to match figures in the main doc. Include the molecular weight ladder.	X
**RNAi, Gene Expression, Microarrays**	At least two different siRNAs targeting different gene areas must be used.	N/A
	At least two different control siRNAs must be used.	N/A
	Gene expression studies cannot be presented alone without providing evidence that the changes in levels have downstream functional consequences.	N/A
	Microarray data must include:<br>○ The raw data for each hybridization.<br>○ Experimental Factors and values.<br>○ Experimental design.<br>○ Data processing protocols (e.g., normalization method).	N/A
**Cell culture**	At least three appropriate cell lines should be used to confirm findings. If there are fewer, add a statement explaining why only 1 or 2 were used.	N/A
